# Intelligent Clustering and Adaptive Energy Management in Wireless Sensor Networks with KDE-Based Deployment

**DOI:** 10.3390/s25082588

**Published:** 2025-04-19

**Authors:** Mainak Kundu, Ria Kanjilal, Ismail Uysal

**Affiliations:** 1 Department of Electrical Engineering, University of South Florida, Tampa, FL 33620, USA; iuysal@usf.edu; 2 Department of Computer Engineering, California Polytechnic State University, San Luis Obispo, CA 93407, USA; rkanjila@calpoly.edu

**Keywords:** wireless sensor networks, kernel density estimation, K-means clustering, Baysian cluster head selection, carrier sense multiple access with collision avoidance

## Abstract

Wireless sensor networks (WSNs) are widely used in IoT, environmental monitoring, and industrial systems, but ensuring energy efficiency, extended network lifetime, and reliable communication under real-world constraints remains challenging. This work proposes a novel clustering framework that integrates kernel density estimation (KDE)-based adaptive node deployment, silhouette-optimized K-means clustering, Bayesian cluster head (CH) selection with Gaussian noise-based energy uncertainty modeling, energy-efficient coverage control, and carrier sense multiple access with collision avoidance-based data transmission. Unlike conventional approaches that rely on fixed clustering and uniform deployments, our framework supports terrain-aware node placement and dynamic CH selection based on residual energy and distance under imperfect sensing conditions. Simulation results demonstrate significant improvements in performance, including over 35% extension in network lifetime and higher coverage retention under energy constraints, compared to baseline methods such as LEACH and K-LEACH. While detailed metrics vary per run due to adaptive parameters and stochastic node behavior, these outcomes validate the scalability, robustness, and practical relevance of the proposed method for real-world WSN deployments.

## 1. Introduction

Wireless sensor networks (WSNs) have garnered substantial research interest due to their wide range of applications in environmental monitoring, healthcare, military surveillance, and industrial automation. One of the primary challenges in WSNs is energy efficiency, as sensor nodes are battery-powered and often deployed in remote or inaccessible locations. To address this challenge, researchers have proposed various clustering-based routing protocols to optimize energy consumption and extend network lifetime. The earliest breakthrough in clustering-based WSNs was introduced by Heinzelman et al., who proposed the low-energy adaptive clustering hierarchy (LEACH) protocol [1]. LEACH introduced a hierarchical clustering approach, where cluster heads (CHs) were randomly elected in each round to evenly distribute the energy load across the network. The incorporation of data aggregation at cluster heads significantly reduces redundant transmissions, improving network efficiency. However, LEACH experiences uneven energy depletion, particularly among CHs, leading to rapid node failures.

To address the limitations of the LEACH protocol, Manjeshwar and Agrawal developed TEEN (threshold-sensitive energy efficient network), which introduced a hard and soft threshold mechanism to regulate data transmission [2]. This mechanism minimizes unnecessary transmissions in time-critical applications, but this is not well-suited for applications requiring periodic data reporting. Building upon LEACH, the authors in [3] introduced PEGASIS (power-efficient gathering in sensor information systems), which replaced the cluster structure with a chain-based formation to minimize transmission distances. PEGASIS effectively extends network lifetime by ensuring that only one node transmits to the base station per round, though it introduces increased latency due to sequential transmissions. Recognizing the need for distributed clustering approaches, another study introduced HEED (hybrid energy-efficient distributed clustering), which used residual energy and communication cost as primary metrics for cluster formation [4]. Unlike LEACH, which randomly selects CHs, HEED ensures a more even energy distribution, leading to better network stability. Around the same time, in [5], the authors developed SEP (stable election protocol), specifically designed for heterogeneous networks with nodes having different energy levels. SEP introduces weighted probabilities for CH selection, extending the lifetime of high-energy nodes and improving overall network stability.

In subsequent years, further refinements had been made to improve the adaptability and efficiency of clustering algorithms. Wang et al. introduced a distributed energy-efficient clustering algorithm, which optimized CH selection by considering both energy levels and network topology [6]. Their method helps maintain load balance and reduce premature node depletion. Meanwhile, El Khediri et al. proposed a K-means and K-medoids clustering-based approach, which leveraged unsupervised learning techniques to form well-balanced clusters, improving both energy consumption and data transmission efficiency [7].

As research progressed, optimization and AI-based methods began influencing WSN clustering. Mahajan and Sharma proposed fuzzy logic-based CH selection, which dynamically adjusted selection criteria based on energy levels and node density, ensuring a more intelligent decision-making process [8]. Despite significant advancements in clustering-based WSN protocols, optimizing energy efficiency, network resiliency, and coverage preservation remains a critical challenge. A study [9] introduced a self-stabilizing lifetime optimization algorithm that dynamically replaced failed nodes to enhance network resilience, particularly in high-density deployments, where redundancy could be exploited. Similarly, in another study [10], the authors proposed an adaptive clustering-based dynamic routing approach using generalized ant colony optimization. By modeling sensor nodes as artificial ants that dynamically select energy-efficient paths, their method significantly improves network lifetime and reliability while reducing transmission overhead. To enhance clustering efficiency, Ghasemzadeh et al. [11] developed BN-LEACH, a Bayesian network-based extension of LEACH that refined CH selection by incorporating distance to the base station, residual energy, and node density. This probabilistic approach ensures a more uniform CH distribution, balancing energy consumption and reducing premature node depletion. Additionally, Shankara et al. [12] introduced a hybrid harmony search algorithm (HSA)–particle swarm optimization (PSO) model, leveraging HSA’s search efficiency and PSO’s adaptability to improve energy-aware CH selection. Their method demonstrates significant improvements in residual energy preservation and throughput, reinforcing the potential of hybrid metaheuristic techniques for WSN optimization.

Beyond clustering and routing, efficient node deployment plays a crucial role in ensuring sustained network performance. In [13], the authors addressed the connected target coverage problem in WSNs for wind farm monitoring by proposing a deterministic relay node deployment algorithm. Their approach utilizes fermat points and convex hull-based relay placement to establish optimal multi-hop connectivity in large-scale deployments. In another study [14], the authors introduced an improved LEACH algorithm, integrating concepts from both LEACH and stable election protocol to optimize CH computation and selection. This approach aims to reduce the mortality rate of sensor nodes by refining the cluster formation process and improving data aggregation before transmission to the base station [14].

Recently, the integration of machine learning and optimization techniques in clustering has become more prevalent. El Khediri et al. proposed an improved K-means clustering algorithm to enhance node localization and cluster formation in WSNs [15]. Their approach considers network topology constraints and employed optimal K-means to ensure a uniform spatial distribution of cluster heads, leading to more balanced energy consumption across the network [15]. Expanding on the potential of unsupervised learning, Tadros et al. introduced an unsupervised learning-based clustering method specifically designed for environmental pollution monitoring [16]. By combining K-means clustering with hierarchical LEACH, their model enables efficient decision making for water-quality monitoring applications, demonstrating its applicability in real-world WSN deployments [16]. The integration of hierarchical clustering techniques further refines energy-efficient WSN protocol. Zeng et al. introduced a threshold-driven K-means sector clustering algorithm, which incorporated symmetrical sector division and energy-aware cluster head selection. Their model achieved balanced cluster sizes, reducing hotspot issues where certain nodes consumed excessive energy due to heavy traffic loads [17]. In another study [18], the authors explored the synergistic integration of K-means clustering with the LEACH protocol, prioritizing nodes with higher residual energy and optimal spatial distribution. Their empirical validation demonstrated significant improvements in network longevity and communication efficiency, setting a benchmark for future WSN clustering strategies [18]. Further refinements in hierarchical clustering methodologies were introduced by proposing LEACH-Kmeans, an enhanced version of LEACH incorporating K-means clustering for more optimal CH selection. This technique significantly reduces energy consumption and improved network lifespan, highlighting the advantages of machine learning-driven clustering over traditional random selection mechanisms [19]. From the early hierarchical approaches like LEACH to modern machine learning-based clustering protocols, the evolution of WSN clustering techniques has focused on optimizing energy consumption, enhancing network scalability, and improving communication efficiency. The adoption of K-means clustering, Bayesian networks, and fuzzy logic has significantly improved CH selection and energy balancing, yet challenges remain in dynamic coverage adaptation and real-time energy-aware clustering.

Our contribution focuses on developing a robust and adaptive clustering framework for WSNs, designed to address challenges such as uneven energy depletion, constrained node placement, and unreliable communication. As a novel approach, we introduce a kernel density estimation (KDE)-based node deployment strategy that enables terrain-aware and spatially adaptive placement, ensuring energy-balanced coverage even under real-world constraints. The objective, to prolong network lifetime, optimize energy usage, and maintain reliable communication, is achieved through a synergy of key components: (1) silhouette-based optimal cluster count selection for balanced clustering, (2) Bayesian cluster head selection combining prior knowledge and real-time energy-distance likelihoods, enhanced with Gaussian noise to model sensing uncertainty, (3) dynamic CH eligibility and adaptive weighting to reflect network energy conditions, (4) cluster viability checks to avoid redundant CH assignment, (5) adaptive coverage radius control to conserve energy, and (6) CSMA/CA (carrier sense multiple access with collision avoidance)-based communication to reduce contention and packet loss.

These integrated mechanisms collectively align with core WSN performance metrics: extended network lifetime through energy efficient CH rotation, improved energy efficiency via optimized placement and adaptive transmission, sustained sensing coverage through residual energy-based radius scaling, and enhanced reliability via contention-aware communication. The proposed framework is not only scalable and lightweight but also adaptable to terrain-constrained deployments, making it well-suited for real-world WSN applications such as environmental monitoring, smart agriculture, and industrial automation. The rest of the paper is organized as follows. Section 2 of this article describes the methodology of the proposed approach. The experimental setup including the initial simulation parameters and the results are presented in Section 3 and Section 4, respectively, followed by Section 5, which provides a detailed discussion of the performance of this novel approach. Finally, Section 6 provides the conclusions and a discussion on potential future work.

## 2. Materials and Methods

### 2.1. Kernel Density Estimation for Optimized Sensor Node Deployment

To address the limitations of random and uniform sensor node deployment in wireless sensor networks, kernel density estimation is introduced as a novel approach for optimizing node placement [20,21]. Unlike traditional methods, KDE provides a data-driven, non-parametric technique for generating an adaptive spatial distribution of sensor nodes. Given a set of *n* independent and identically distributed data points $x_{1},x_{2},\ldots ,x_{n}$, KDE estimates the density function using the expression

(1) $$\hat{f}\left(x\right)=\frac{1}{n}\sum_{i=1}^{n}K_{h}\left(x_{i},t\right),$$

where $K_{h}\left(x,t\right)$ is the kernel function that defines the contribution of each sample point to the estimated density, *h* is the bandwidth parameter controlling the smoothness of the estimated density, $x_{i}$ are the sample points, and *n* is the number of sample points. Bandwidth selection plays a critical role in KDE, as it determines the trade-off between excessive smoothing and overfitting. In this implementation, an adaptive bandwidth selection method is employed using the leave-one-out cross-validation (LOO-CV) technique to achieve optimal density estimation. Given a dataset *X*, a candidate bandwidth *h*, and a kernel function *K*, the LOO-CV function iteratively removes a single data point, estimates the density function using the remaining data, and evaluates the likelihood of the excluded point. The log-likelihood scores are accumulated, and the total negative log-likelihood (NLL) is computed to quantify the goodness of fit, which is represented as

(2) $$NLL\left(h\right)=-\sum_{i=1}^{n}logf_{-i}\left(x_{i}\right),$$

where $f_{-i}\left(x_{i}\right)$ is the estimated density at $x_{i}$ without the *i*-th point. The optimal bandwidth is selected by minimizing the NLL over a predefined range, ensuring accurate probability density function (PDF) estimation while avoiding excessive smoothing. Once determined, KDE is utilized to generate the sensor node distribution by constructing a grid of spatial points and computing density values using the radial kernel estimator, which is shown as

(3) $$\hat{f}\left(x,y\right)=\frac{1}{n}\sum_{i=1}^{n}K\left(\frac{{\left(x-x_{i}\right)}^{2}+{\left(y-y_{i}\right)}^{2}}{h^{2}}\right),$$

where (x,y) are the coordinates of the estimation points, $\left(x_{i},y_{i}\right)$ represent the spatial coordinates of the deployed sensor nodes used as input for the kernel density estimation, and *h* is the bandwidth smoothing parameter. This approach is particularly suited for geospatial analysis, as it effectively captures spatial dependencies by incorporating euclidean distance-based smoothing.

### 2.2. K-Means Clustering with Optimal Cluster Count

In the proposed method, K-means clustering [22] is applied after determining the optimal number of clusters ($k^{*}$), ensuring balanced cluster formation and efficient energy utilization. Instead of dynamically adjusting the number of clusters in each round, we determine $k^{*}$ before clustering and apply K-means to partition the network into equal-sized clusters. To determine the optimal cluster count ($k^{*}$), we utilize the silhouette score method, which evaluates clustering quality by analyzing how well nodes are grouped within clusters. For each candidate cluster count *k*, the corresponding silhouette score $S_{k}$ is computed as [23,24]

(4) $$S_{k}=\frac{b(i)-a(i)}{\max(a(i),b(i))},\;\;$$

where $S_{k}$ is the silhouette score for a given number of clusters *k*, a(i) is the average intra-cluster distance, which measures how close a node is to other nodes within the same cluster, and b(i) is the average inter-cluster distance, which measures how close a node is to the nearest cluster. The optimal number of clusters $k^{*}$ is determined using the silhouette score $S_{k}$ is maximized as

(5) $$k^{*}=\arg\max_{k_{\min}\leq k\leq k_{\max}}S_{k},$$

where $S_{k}$ is the silhouette score computed for different values of *k*.

### 2.3. Applying K-Means Clustering

Once the optimal cluster count is determined, K-means clustering is applied to partition the sensor nodes into $k^{*}$ clusters. The algorithm aims to minimize intra-cluster variance, represented mathematically as [25,26,27]

(6) $$J=\sum_{i=1}^{n}\sum_{k=1}^{k^{*}}\delta_{ik}\left|\right|x_{i}-\mu_{k}{\left|\right|}^{2},$$

where $\mu_{k}$ represents the centroid of cluster *k*, and $k^{*}$ denotes the precomputed optimal number of clusters. In (6), $\delta_{ik}=1$ if node $x_{i}$ belongs to cluster *k*; otherwise, it is 0. Each sensor node $x_{i}$ is assigned to the nearest centroid $\mu_{k}$ based on the euclidean distance, given by

(7) $$d\left(x_{i},\mu_{k}\right)=\sqrt{{\left(x_{i1}-\mu_{k1}\right)}^{2}+{\left(x_{i2}-\mu_{k2}\right)}^{2}}\;.$$

Once the clusters are formed, the centroids are updated by recalculating their positions as the mean of all member nodes defined as

(8) $$\mu_{k}=\frac{1}{|C_{k}|}\sum_{x_{i}\in C_{k}}x_{i},$$

where $C_{k}$ is the set of nodes belonging to cluster *k*. This centroid adjustment process continues until it converges.

### 2.4. Bayesian Cluster Head Selection with Energy Uncertainty Modeling

In each cluster, sensor nodes rely on a designated CH for data aggregation and forwarding to the sink. To improve energy efficiency and extend network longevity, we employ a dynamic Bayesian-based CH selection strategy. This probabilistic approach integrates both prior knowledge and real-time network observations to ensure fair, energy-aware CH election.

In real-world WSNs, residual energy measurements are often imperfect due to sensing noise, delayed updates, or intermittent communication failures. To realistically capture this behavior, our Bayesian CH selection model incorporates a lightweight stochastic mechanism by injecting small Gaussian noise into the residual energy values used during CH ranking. Specifically, for each node *i*, the perceived energy used in the likelihood computation is modeled as

(9) $${\tilde{E}}_{i}=E_{i}+\epsilon_{i},\;\epsilon_{i}∼N\left(0,\sigma^{2}\right),$$

where $E_{i}$ is the true residual energy of node *i*, $\epsilon_{i}$ is Gaussian noise with zero mean and small variance $\sigma^{2}$, and ${\tilde{E}}_{i}$ is the noisy energy used only for CH ranking. In our implementation, we set σ=0.001 to reflect lightweight perception error without significantly distorting the energy distribution. To preserve physical realism, the noisy energy values are clipped within the range $\left[0,E_{0}\right]$. $E_{0}$ refers to the initial energy level assigned to each node, previously defined as a constant value ($E_{0}=0.5$ J).

Importantly, this noise is applied only during the likelihood evaluation step and does not influence actual energy consumption, node death, or CH eligibility threshold checks. These operations continue to use the true (noiseless) residual energy, ensuring simulation correctness while improving decision robustness. The likelihood-based CH selection probability for a node *i* is computed as

(10) $$P_{CH}\left(i\right)=\alpha {\left(\frac{{\tilde{E}}_{i}}{E_{0}}\right)}^{2}+\left(1-\alpha \right)\left(\frac{1}{1+d_{i}}\right),$$

where $d_{i}$ is the Euclidean distance between the node and the sink, and α is a dynamic weight factor controlling the relative importance of energy versus distance. To avoid overburdening nodes with insufficient energy, we assign $P_{CH}\left(i\right)=0$ for all $E_{i}<E_{\mathrm{threshold}}$, using the true energy $E_{i}$ for this eligibility condition. The Bayesian posterior probability for each node is then updated as

(11) $$P_{\mathrm{posterior}}\left(i\right)=\alpha P_{\mathrm{prior}}\left(i\right)+\left(1-\alpha \right)P_{\mathrm{likelihood}}\left(i\right),$$

where $P_{\mathrm{prior}}\left(i\right)$ represents the prior probability (uniform if not previously defined), and $P_{\mathrm{likelihood}}\left(i\right)$ is computed based on the noisy energy ${\tilde{E}}_{i}$ and sink distance.

Moreover, the weight factor α is dynamically adjusted based on the average residual energy in the network. In early rounds, when energy estimates are relatively accurate and node health is stable, the model favors the likelihood component. In later rounds, when energy depletes and node state becomes more uncertain, the algorithm shifts weight toward prior beliefs. This adaptive behavior improves resilience against measurement noise and network degradation, ensuring robust and context-aware CH selection.

### 2.5. CSMA/CA for Efficient Data Transmission

In the transmission phase, each node gathers data and transmits it to its CH using the carrier sense multiple access with collision avoidance protocol to reduce collisions and improve energy efficiency. In this approach, each node first senses the channel. If the channel is idle, it transmits immediately. If the channel is busy, it initiates a randomized backoff mechanism before retrying.

**Figure 1:**
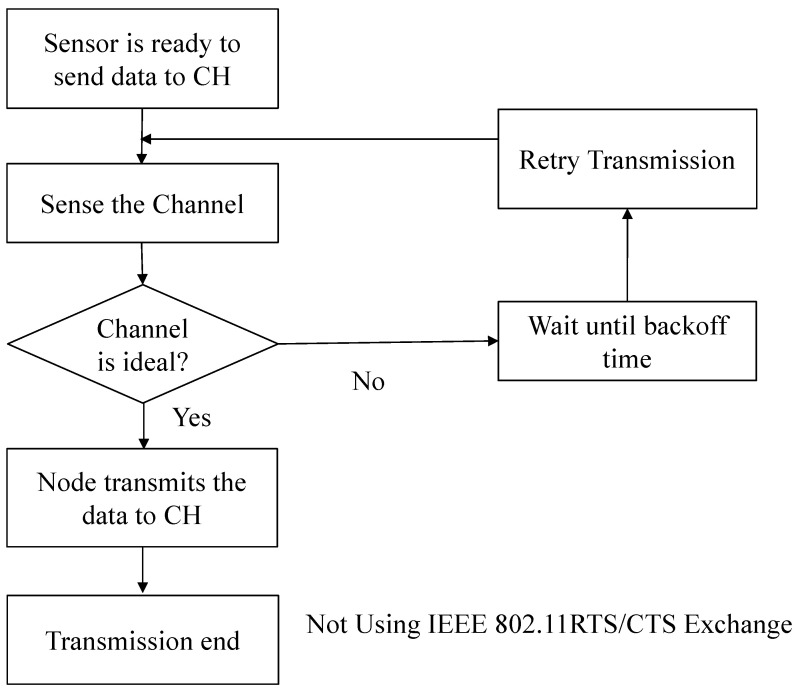
Flowchart of data transmission using CSMA/CA.

In our implementation, the backoff duration is abstracted probabilistically: each node selects a random integer between 1 and a maximum backoff window size, and attempts transmission with a success probability inversely proportional to this value. This simulates a contention-based wait period where nodes experiencing congestion are less likely to retry immediately, thereby reducing the chance of repeated collisions. Unlike CSMA/CD [28], which detects collisions after they occur, CSMA/CA proactively avoids collisions, making it better suited for wireless environments like WSNs [29]. Figure 1 illustrates the CSMA/CA-based data transmission process, which enhances reliability and energy conservation in our proposed framework.

### 2.6. Energy Consumption Modeling

Each sensor node communicates via a designated cluster head, responsible for aggregating and relaying data to the sink. Communication occurs in two phases: (1) intra-cluster communication, where member nodes transmit data to their respective CHs, and (2) inter-cluster communication, where CHs forward aggregated data to the sink. The energy dissipation for a sensor node *i* transmitting a packet of size *k* bits over a distance *d* is represented as

(12) $$E_{TX}\left(k,d\right)=\left\{\begin{array}{cc} kE_{TX}+kE_{fs}d^{2}, & d\leq d_{0}\\ kE_{TX}+kE_{mp}d^{4}, & d>d_{0}, \end{array}\right.$$

where $E_{fs}$ and $E_{mp}$ are the free-space and multi-path fading energy coefficients, respectively. In (12), $d_{0}$ is the threshold distance, which is denoted as

(13) $$d_{0}=\sqrt{\frac{E_{fs}}{E_{mp}}}\;.\;\;$$

Each CH aggregates the received data and transmits it to the base station. The energy required for data aggregation is represented as

(14) $$E_{DA}=kE_{DA},$$

where $E_{DA}$ is the energy required for data fusion per bit.

### 2.7. Simulation Rounds with Dynamic CH Selection

The proposed dynamic CH selection mechanism enhances energy efficiency and adaptive clustering in WSNs by assigning CHs based on residual energy and transmission cost. Nodes first verify CH availability, selecting only those above the energy threshold; if none remain, they refrain from cluster association. The algorithm removes depleted CHs and prioritizes those minimizing transmission cost, considering distance and the appropriate model (free-space or multi-path). By favoring energy-efficient CHs, this adaptive strategy extends network longevity and ensures reliable communication. CHs are continuously monitored, and once below the threshold, new CHs are selected using a Bayesian probabilistic approach, optimizing energy consumption and network performance.

### 2.8. Dynamic Coverage Radius Adaptation

In this study, the coverage radius of sensor nodes is dynamically adjusted based on their residual energy to optimize sensing efficiency and prolong network lifetime. Initially, when all the sensor nodes have their maximum initial energy $E_{0}$, the initial coverage radius for each node is given by

(15) $$R_{i}^{initial}=R_{max}\;.$$

As nodes participate in data transmission, their energy depletes based on the number of transmitted bits and the associated energy consumption per bit for transmission and reception. To account for energy depletion, the dynamically computed coverage radius is continuously updated using (16)

(16) $$R_{i}=R_{min}+\left(R_{max}-R_{min}\right)\left(\frac{E_{i}-E_{threshold}}{E_{0}-E_{threshold}}\right)\;,$$

where $E_{i}=E_{0}\;exp\left(-\lambda_{i}\right)$, $E_{i}$ represents the residual energy at round *i*, λ is a decay factor, and $R_{min}$ ensures that nodes maintain a minimum sensing range even at lower energy levels. Nodes with higher residual energy sustain a larger coverage area, while those with reduced energy gradually shrink their sensing range, leading to an adaptive coverage pattern. Additionally, when a node’s energy drops below a predefined threshold $E_{threshold}$, it is considered dead and removed from coverage calculations. This adaptive mechanism prevents unnecessary energy depletion while balancing network-wide coverage.

**Figure 2:**
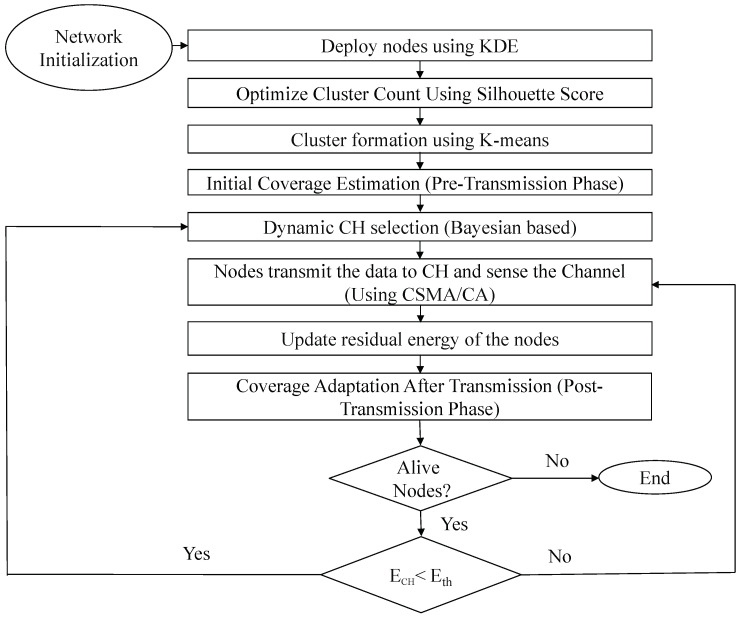
Flowchart of the proposed method.

The flowchart in Figure 2 outlines the step-by-step process of the proposed method. It begins with network initialization and node deployment using KDE, followed by optimizing the number of clusters through the silhouette score. K-means clustering is then applied for cluster formation, and CHs are selected using a Bayesian-based approach and CHs change dynamically based on residual energy during the rounds of operation. Sensor nodes transmit data to their respective CH while sensing the channel using CSMA/CA. After transmission, residual energy is updated, and the coverage area is updated accordingly. The process continues iteratively until no alive nodes remain in the network.

## 3. Experimental Setup

The simulation was conducted using the following parameters as mentioned in Table 1, ensuring a realistic WSN environment. Moreover, to make a fair comparison with other results, we considered the same initial parameter setup. The network consists of 100 sensor nodes deployed within a 100 m × 100 m area with a centralized sink.

## 4. Results

**Figure 3:**
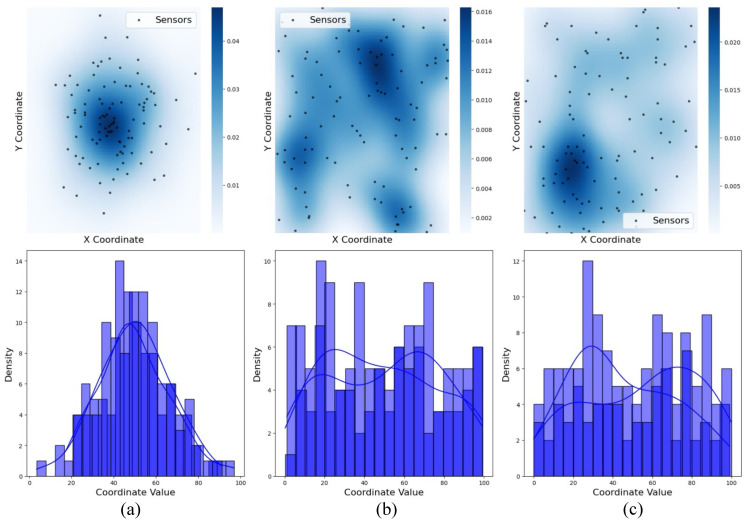
Sensor node deployment strategies: heatmap and histogram plots for (**a**) Gaussian distribution, (**b**) random distribution, and (**c**) KDE-based distribution. Heatmaps display estimated node density, with darker regions indicating higher concentrations. Colorbar values represent unitless, normalized probability densities derived using KDE over the 2D spatial domain (0–100 units).

Figure 3 illustrates the impact of different sensor deployment strategies on network coverage and energy efficiency. Random uniform deployment ensures an even spatial distribution but lacks density optimization, leading to coverage gaps and uneven energy consumption [1]. These inconsistencies affect the cluster formation and overall network performance. Gaussian-based deployment, which results in a higher concentration of nodes near the center, improves connectivity near the sink [30]. However, it also causes coverage redundancy and localized energy depletion. In contrast, the KDE-based deployment optimizes sensor placement using probabilistic density estimation, ensuring balanced coverage and energy distribution. This enhances network longevity, making it a more effective deployment strategy for sustainable WSN operations. The heatmaps use a color gradient from pale to dark blue to represent the estimated sensor node density, where darker regions indicate higher concentrations of nodes.

**Figure 4:**
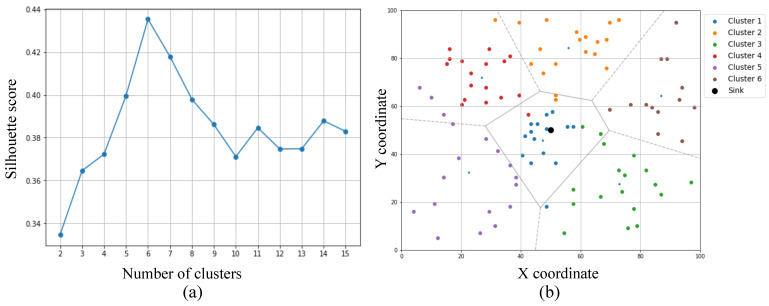
Cluster formation following sensor deployment: (**a**) the silhouette score for varying cluster counts and (**b**) cluster formation based on the optimal number of clusters corresponding to the highest silhouette score in the first panel.

The performance of the proposed KDE-based clustering framework was evaluated over 5000 simulation rounds using 100 sensor nodes deployed via kernel density estimation. This adaptive deployment strategy provides spatial balance by aligning node placement with estimated spatial density distributions, unlike conventional uniform or random deployments. For this particular simulation, the optimal KDE bandwidth was automatically selected as 10.61 m using leave-one-out cross-validation. Based on silhouette score analysis, the optimal number of clusters was determined to be 6, as illustrated in Figure 4a, with the resulting cluster structure shown in Figure 4b. Due to stochastic factors such as KDE sampling and K-means initialization, both the selected bandwidth and number of clusters may vary across different runs, demonstrating the method’s flexibility in adapting to spatial conditions and distinguishing it from traditional K-means clustering, which fixes the number of clusters a priori.

**Figure 5:**
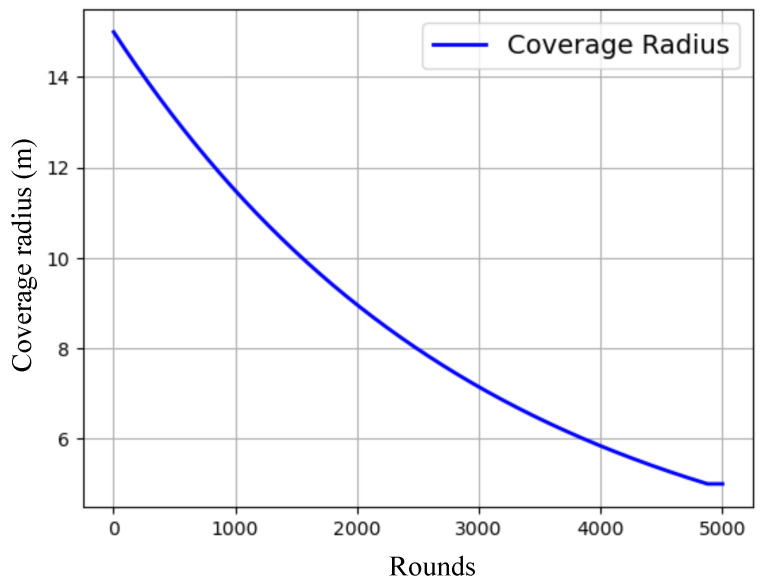
Dynamic coverage radius over rounds.

**Figure 6:**
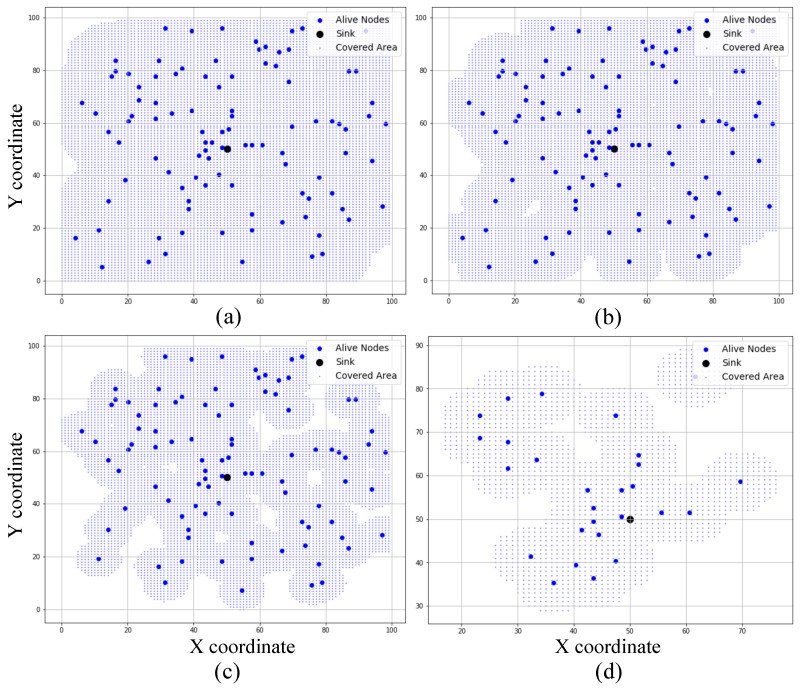
Network coverage area across different simulation rounds: (**a**) 97.76% coverage at round 1, (**b**) 91.77 % coverage at round 2500, (**c**) 73.86 % coverage at round 4000, and (**d**) 20.39 % coverage at round 4500.

**Figure 7:**
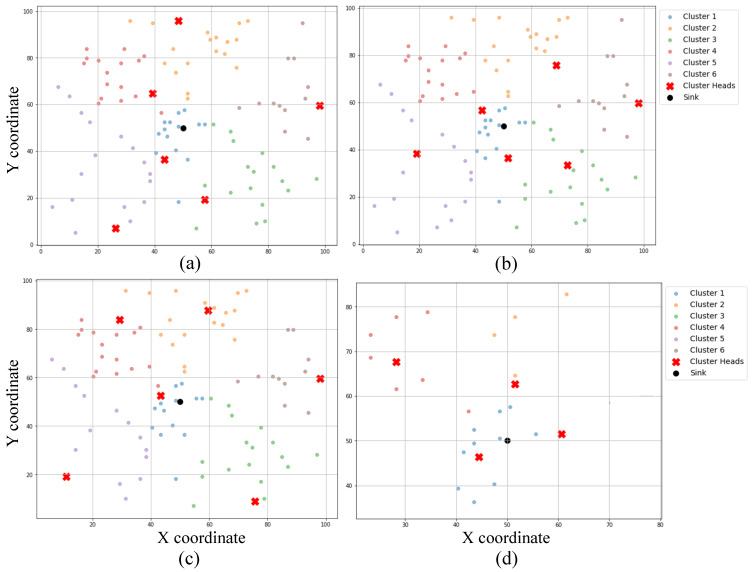
Cluster head selection at different simulation rounds: (**a**) round 1, (**b**) round 2500, (**c**) round 4000, and (**d**) round 4500.

**Figure 8:**
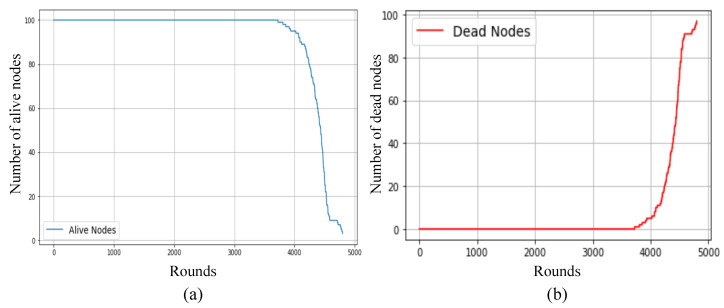
Alive and dead node analysis over simulation rounds: (**a**) number of alive nodes over rounds and (**b**) number of dead nodes over rounds.

**Figure 9:**
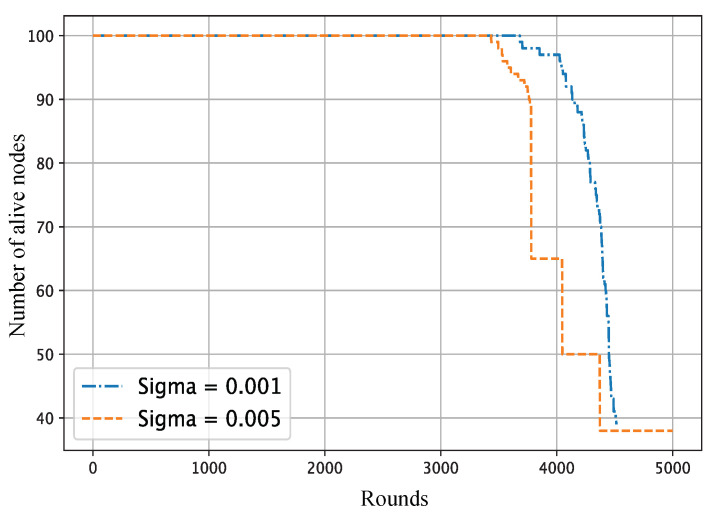
Comparison of the number of alive nodes over simulation rounds for different sigma values.

Figure 5 presents the gradual decline in each node’s coverage radius in response to residual energy, and Figure 6 demonstrates how this affects the overall network coverage area, decreasing from 97.76% at round 1 to just 20.39% at round 4500. Figure 7 highlights the temporal progression of CH locations, while Figure 8 illustrates the trends in alive and dead node counts throughout the simulation. All 100 nodes remained alive and active until round 3754, with the first node death recorded at round 3755. From round 4000 onward, the rate of node failure gradually increased and intensified further after round 4500. At round 4500, the network retained 27 operational nodes, which were dynamically organized into four active clusters, each led by a functioning cluster head. This confirms the framework’s ability to maintain network operability and adaptively restructure clusters under energy-constrained conditions. The simulation terminated at round 4799, when no eligible nodes remained to form viable cluster heads. Although three nodes were still technically alive at round 5000, all had residual energy levels below the threshold required for communication, rendering them non-functional from a network perspective. The integration of KDE-based deployment, Bayesian CH selection with energy uncertainty modeling, adaptive coverage, and CSMA/CA communication collectively contributed to a significant extension in network lifetime, improved energy balance, and more resilient connectivity under practical WSN constraints. Moreover, to evaluate the impact of sensing uncertainty on network degradation, we experimented and simulated the proposed Bayesian CH selection model under two different standard deviations (σ=0.001 and σ=0.005) of Gaussian noise applied to residual energy perception. As shown in Figure 9, higher noise levels accelerated node death, with σ=0.005 leading to earlier and steeper increases in dead node count, thereby demonstrating the sensitivity of WSN performance to energy estimation fidelity.

**Figure 10:**
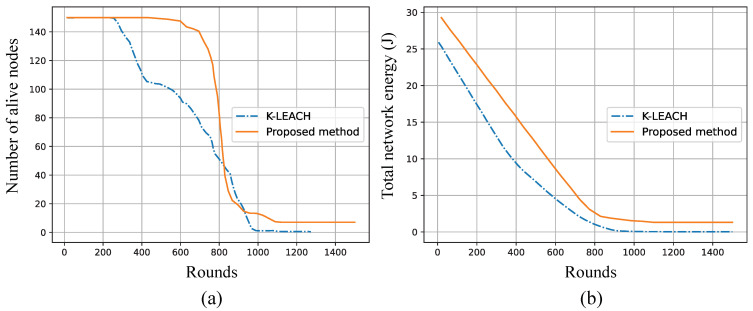
Comparison of (**a**) the number of alive nodes and (**b**) total network energy over simulation rounds between K-LEACH [18] and the proposed method.

**Figure 11:**
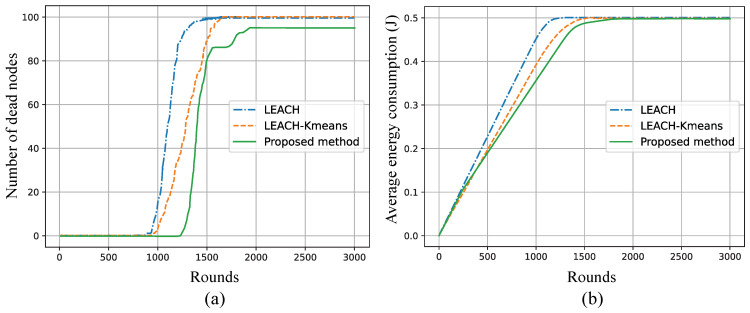
Comparison of (**a**) the number of dead nodes and (**b**) average energy consumption over simulation rounds between LEACH, LEACH-Kmeans [19], and the proposed method.

To ensure a fair comparison with state-of-the-art methods, we evaluated the simulation results of our proposed approach against LEACH [19] and K-means-based clustering techniques, including K-LEACH [18] and LEACH-Kmeans [19]. The simulations were conducted using the same parameters to perform a consistent and unbiased comparison. As observed in Figure 10, our proposed method consistently outperforms K-LEACH in terms of node survival over simulation rounds, maintaining a higher number of alive nodes for an extended period, which indicates better energy management and prolonged network lifetime. Unlike K-LEACH, which relies on K-means clustering for cluster head selection, the proposed method dynamically adapts the cluster formation based on KDE, optimizing coverage while ensuring balanced energy consumption among nodes. Similarly, Figure 11 illustrates the comparison with LEACH and LEACH-Kmeans, where the proposed method exhibits superior energy conservation and a slower rate of node depletion. While LEACH suffers from random CH selection, and LEACH-Kmeans improves stability through structured clustering, neither fully accounts for adaptive energy-based coverage adjustments, which is a key strength of the proposed method. The results demonstrate that this proposed method achieves enhanced energy efficiency and extended network lifespan compared to the traditional and K-means-based clustering methods, making it a more robust solution for energy-constrained wireless sensor networks.

## 5. Discussion

The proposed KDE-based clustering framework offers a practical and scalable solution for enhancing WSN performance by integrating spatially optimized node deployment, adaptive clustering, and energy-efficient communication. One of the key innovations is the use of kernel density estimation (KDE) for terrain-adaptive sensor deployment. Unlike random or uniform placement strategies, KDE allows nodes to be distributed based on real-world constraints such as terrain features, accessibility, and application-specific coverage priorities. This results in more balanced energy consumption, reduced hotspots, and improved overall coverage efficiency. The framework’s flexibility also makes it suitable for mission-driven WSN applications such as environmental monitoring, smart agriculture, and urban sensing.

On the clustering side, we incorporate a Bayesian cluster head (CH) selection mechanism that adapts to residual energy and node location. To account for imperfect energy sensing, caused by noise or communication delays, a small Gaussian noise is added during CH ranking, allowing the algorithm to model uncertainty realistically without affecting energy depletion logic. Additionally, the decision weight factor α is dynamically adjusted based on the network’s average energy level, balancing the influence of prior probabilities and real-time observations throughout the simulation. Cluster viability is further ensured by filtering out inactive clusters using a functional node check, which prevents CH assignment in depleted or isolated regions.

For communication, CSMA/CA is employed to enable collision avoidance through randomized backoff. This improves transmission reliability, reduces packet loss, and reflects the realities of shared wireless channels. To conserve energy further, coverage radii are adjusted dynamically based on each node’s remaining energy, enabling high-energy nodes to maintain wider sensing ranges while low-energy nodes reduce their load.

These components collectively contribute to four core performance metrics emphasized in WSN research: extended network lifetime, improved energy efficiency, sustained coverage, and reliable communication. Network lifetime is significantly prolonged by dynamically selecting cluster heads based on residual energy and distance, thereby preventing premature node failure. Energy efficiency is achieved through optimal node placement, minimal intra-cluster transmission distances, and adaptive sensing radii, all of which reduce unnecessary energy expenditure. The adaptive coverage model maintains sensing effectiveness across the network lifespan by proportionally adjusting each node’s coverage based on its remaining energy. Meanwhile, communication reliability is improved by incorporating CSMA/CA during data transmission, which reduces channel contention and minimizes the risk of packet collisions in a wireless medium. The simulation results of the proposed method demonstrate that the network maintains connectivity longer, minimizes energy waste, and preserves sensing coverage more effectively than baseline approaches. Additionally, the simulation halts automatically when no CHs can be elected, either due to complete energy depletion or a lack of viable clusters, reflecting realistic network shutdown conditions where nodes, although still alive, can no longer sustain communication.

From a computational standpoint, the framework remains lightweight. KDE-based node deployment and cluster count optimization are performed once with $O(n^{2})$ complexity. Each simulation round involves CH selection, CSMA/CA logic, and energy updates with a practical complexity of O(n·k), which is effectively linear when *k* is small. The full simulation (100 nodes, 5000 rounds) runs in approximately 110 s on a standard workstation (Intel(R) Core(TM) i5-8265U CPU @ 1.60 GHz, 8 GB RAM), confirming the framework’s efficiency and scalability.

## 6. Conclusions

This study explored an optimized clustering framework for WSNs, integrating KDE-based node deployment, K-means clustering, Bayesian CH selection with energy uncertainty modeling, adaptive coverage control, and CSMA/CA-based data transmission to address key challenges in energy efficiency and network longevity. The KDE-based deployment ensures balanced node distribution, reducing coverage gaps and enhancing energy utilization. K-means clustering efficiently partitions the network into well-balanced clusters, while Bayesian CH selection dynamically adapts to real-time energy conditions, preventing premature CH depletion. The adaptive coverage mechanism further optimizes energy usage by dynamically adjusting sensor coverage based on residual energy levels. Simulation results confirm significant improvements in network lifetime, coverage retention, and energy efficiency compared to traditional clustering approaches. This framework offers a scalable, energy-aware, and computationally efficient solution for WSNs in IoT, environmental monitoring, and industrial automation. Future research will focus on incorporating machine learning-based adaptive clustering and real-time optimization techniques to further enhance performance in dynamic environments.

## Figures and Tables

**Table 1:** Initial simulation parameters.

Parameter	Value
Number of nodes (*n*)	100
Network area	100 m × 100 m
Sink position	(50, 50)
Initial energy ($E_{0}$)	0.5 J
Transmission energy ($E_{TX}$)	50 nJ/bit
Reception energy ($E_{RX}$)	50 nJ/bit
Free-space energy ($E_{fs}$)	10 pJ/bit/$m^{2}$
Multi-path energy ($E_{mp}$)	0.0013 pJ/bit/$m^{4}$
Threshold energy ($E_{th}$)	$0.2\times E_{0}$, $0.5\times E_{0}$
Message size (*k*)	2000 bits, 4000 bits
Threshold distance ($d_{0}$)	$\sqrt{E_{fs}/E_{mp}}$
Maximum rounds	1500, 3000, 5000
Energy aggregation ($E_{DA}$)	5 nJ/bit/message
Maximum coverage radius ($R_{max}$)	15 m
Minimum coverage radius ($R_{min}$)	5 m

## Data Availability

The data supporting the findings of this study were generated through simulation experiments and data sharing is not applicable to this article. The simulation framework and parameters are described in detail in the manuscript to ensure reproducibility.
